# Quantifying left ventricular myocardial strain in patients with different CAD-RADS levels based on computed tomography feature tracking technology

**DOI:** 10.1038/s41598-023-44530-8

**Published:** 2023-10-11

**Authors:** Na Li, Lijie Zhang, Hongying Wu, Jia Liu, Yukun Cao, Yumin Li, Jie Yu, Xiaoyu Han, Guozhu Shao, Ming Yang, Jin Gu, Lina Chen, Jiangtao Wang, Heshui Shi

**Affiliations:** 1 Department of Radiology, Union Hospital, Tongji Medical College, Huazhong University of Science and Technology, 1277 Jiefang Rd., Wuhan, 430022 China; 2 Hubei Province Key Laboratory of Molecular Imaging, Wuhan, 430022 China; 3 CT Collaboration, Siemens Healthineers Ltd, Guangzhou, 510620 China; 4 Department of Radiology, Xiangyang Central Hospital, Hubei University of Arts and Science, 136 Jingzhou Rd, Xiangyang, 441021 China

**Keywords:** Cardiology, Diagnostic markers

## Abstract

To evaluate myocardial strain in patients with different coronary artery disease-reporting and data system (CAD-RADS) levels using the computed tomography (CT) feature tracking technology and to investigate the relationship of myocardial strain with coronary artery calcium scores (CACs) and the degree of coronary artery stenosis. We prospectively enrolled 237 consecutive patients to undergo coronary CT angiography. The participants were divided into the following groups: control (n = 87), CAD-RADS 1 (n = 43), CAD-RADS 2 (n = 43), CAD-RADS 3 (n = 38), and CAD-RADS 4 and above (n = 26). Myocardial strains were analyzed by commercial software, and CACs and coronary stenosis were assessed on post-processing stations. Differences between multiple groups were analyzed using one-way analysis of variance or the Kruskal–Wallis test. Logistic regression were used to analyze the effects of dichotomous variables. As the CAD-RADS level increased, the global circumferential strain (GCS), global longitudinal strain (GLS) and global radial strain (GRS) of the left ventricle based on CT gradually decreased. A significant correlation was observed between global myocardial strain and CACs (GRS: r =  − 0.219, GCS: r = 0.189, GLS: r = 0.491; *P* < 0.05). The independent predictors of obstructive CAD were age (*β* = 0.065, odds ratio [*OR*] = 1.067, *P* = 0.005), left ventricular ejection fraction (*β* = 0.145, *OR* = 1.156, *P* = 0.047), and GLS (*β* = 0.232, *OR* = 1.261, *P* = 0.01). CT-derived GLS of the left ventricle is correlated with CAD-RADS levels and CACs. It may be a better indicator than CACs to reflect the severity of CAD.

## Introduction

Coronary artery disease (CAD) is a major cardiovascular disease in many countries worldwide. In 2016, the American Heart Association published an updated heart disease and stroke statistics report, which stated that 15.5 million people over the age of 20 in the United States have CAD^1^. Coronary artery calcium score (CACs) is well known as an independent predictor of future coronary events. Quantifying CACs can aid in the prediction of future cardiovascular disease, and an increased CACs is directly related to increased disease risk^2^. The value of myocardial strain for diagnosis and risk stratification has been demonstrated in various cardiovascular diseases, such as severe aortic stenosis^3^, aortic valve replacement^4,5^, adult congenital heart disease^6^, isolated left anterior descending coronary stenosis^7^, and myocardial infarction^8^.

Myocardial feature tracking is a highly reproducible method for measuring myocardial strain^9^. Echocardiography and cardiac magnetic resonance (CMR) are currently used to quantify local systolic cardiac function^10,11^. Due to the angle dependence, sound window limitation, and manipulator dependence of echocardiography and the long acquisition time of CMR, cardiac computerized tomography (CT) has become a rapid, valuable method for assessing myocardial strain and is in good agreement with echocardiography and CMR^3,12,13^.

Therefore, this study aimed to evaluate the differences in CT-derived myocardial strain in patients with different CAD-reporting and data system (CAD-RADS) levels and investigate the relationship of cardiac CT-based myocardial strain with coronary artery calcium scores (CACs) and the degree of coronary stenosis.

## Materials and methods

### Study populations

The study was approved by the ethics committee. Patients with suspected CAD (i.e., patients with symptoms of chest tightness and chest pain) were prospectively enrolled to undergo coronary CT angiography (CTA) to rule out CAD in the Union Hospital in Wuhan from August to November 2020. The CAD-RADS classification was implemented as described in the Cardiology of the American Expert Consensus document^14^; based on the CAD-RADS level, the patients were divided into groups I (CAD-RADS 1), II (CAD-RADS 2), III (CAD-RADS 3), and IV (CAD-RADS 4 and 5). Patients with evidence of arrhythmia, valvular heart disease, cardiomyopathy, congenital heart disease, or poor image quality or those with a history of coronary reconstruction were excluded. The inclusion criteria for the control group were as follows: (1) no history of CAD (≥ 1% diameter stenosis assessed via CTA), cardiomyopathy, valvular heart disease, or other cardiovascular diseases, (2) no hypertension, diabetes, or dyslipidemia, and (3) normal electrocardiograph (ECG) findings obtained 2 weeks before CTA. All subjects with renal dysfunction (glomerular filtration rate < 30 mL/min/1.73 m^2^), pregnant women, and children were excluded.

Data on medical comorbidities were obtained from patients through questionnaires. Hypertension was defined as systolic blood pressure > 140 mmHg, diastolic blood pressure > 90 mmHg, or use of antihypertensive medication. Diabetes was defined as the 2-h plasma glucose > 200 mg/dL during an oral glucose tolerance test (OGTT), or use of glucose-lowering medication. Dyslipidaemia was defined as total cholesterol > 5.7 mmol/L, low-density lipoprotein cholesterol > 4.1 mmol/L, triglyceride > 1.7 mmol/L, or use of cholesterol-lowering drugs. Non-smokers were defined as having less than 100 lifetime cigarettes, and the rest of the patients were categorized as regular smokers. Patients who drank alcohol were defined as having a positive history of alcohol consumption^7^. Family history of CAD was defined as a confirmed diagnosis of CAD in an immediate family member. Patients with incomplete clinical information were excluded from the analysis.

### Cardiac CT acquisition

Cardiac CT was performed using the Siemens third-generation dual-source CT scanner (Somatom Force, Siemens Healthineers, Forchheim, Germany). Patients with heart rates above 90 bpm received 25–50 mg metoprolol (AstraZeneca AB, Sweden) to control heart rate and then, took nitroglycerin (0.5 mg, Shandong Province, China) orally to dilate coronary arteries 3–5 min before examination. Participants were trained to inhale and hold their breath before the examination. The scanning was initiated from the calcification score with a tube voltage of 120 kVp and a layer thickness of 3 mm. Scan range from 1–2 cm below the tracheal bifurcation to the diaphragmatic level of the heart. The retrospective ECG-gated coronary CTA scanning parameters were as follows: detector collimation, 192 × 0.6 mm; gantry rotation time, 0.25 s/r; pitch, 0.15; and slice, 0.75 mm. Automatic tube voltage technology (Care kV, Siemens Healthineers) and intelligent tube current scanning technology (Care Dose 4D, Siemens Healthineers) were used to automatically determine tube voltage and tube current, respectively. Based on the weight of the participants, a total of 30–60 mL of iopromide (400 mg I/mL, Bracco, Patheon Italia S. P. A, Italy) was injected continuously into the median cubital vein at a rate of 2–4 mL/s, followed by an injection of saline solution. 20-phase images were reconstructed in 5% steps of the RR interval. The reconstruction parameters were as follows: thickness, 0.75 mm; the increment, 0.5 mm; reconstruction kernel, Bv40. The effective radiation dose was 4.9 ± 1.4 mSv, which was obtained by multiplying the dose-length product by 0.014.

### CMR acquisition

Eleven patients were randomly selected from the disease groups to undergo CMR using the 3.0 T system (SIEMENS Skyra, Siemens Healthcare, Germany). They were repeatedly trained to inhale and hold their breath to ensure cooperation with commands during examination. A balanced steady-state free precession sequence and retrospective ECG gating were used to acquire left ventricular (LV) long-axis (4-, 3-, and 2-chamber) and short-axis cine images covering the entire LV layer. The parameters were as follows: field of view, 340 mm × 287 mm, matrix, 256 × 216, repetition time/echo time, 34.2 ms/1.53 ms, reversal angle, 90°, and slice thickness, 8 mm.

### CT data postprocessing and analysis

**Figure 1:**
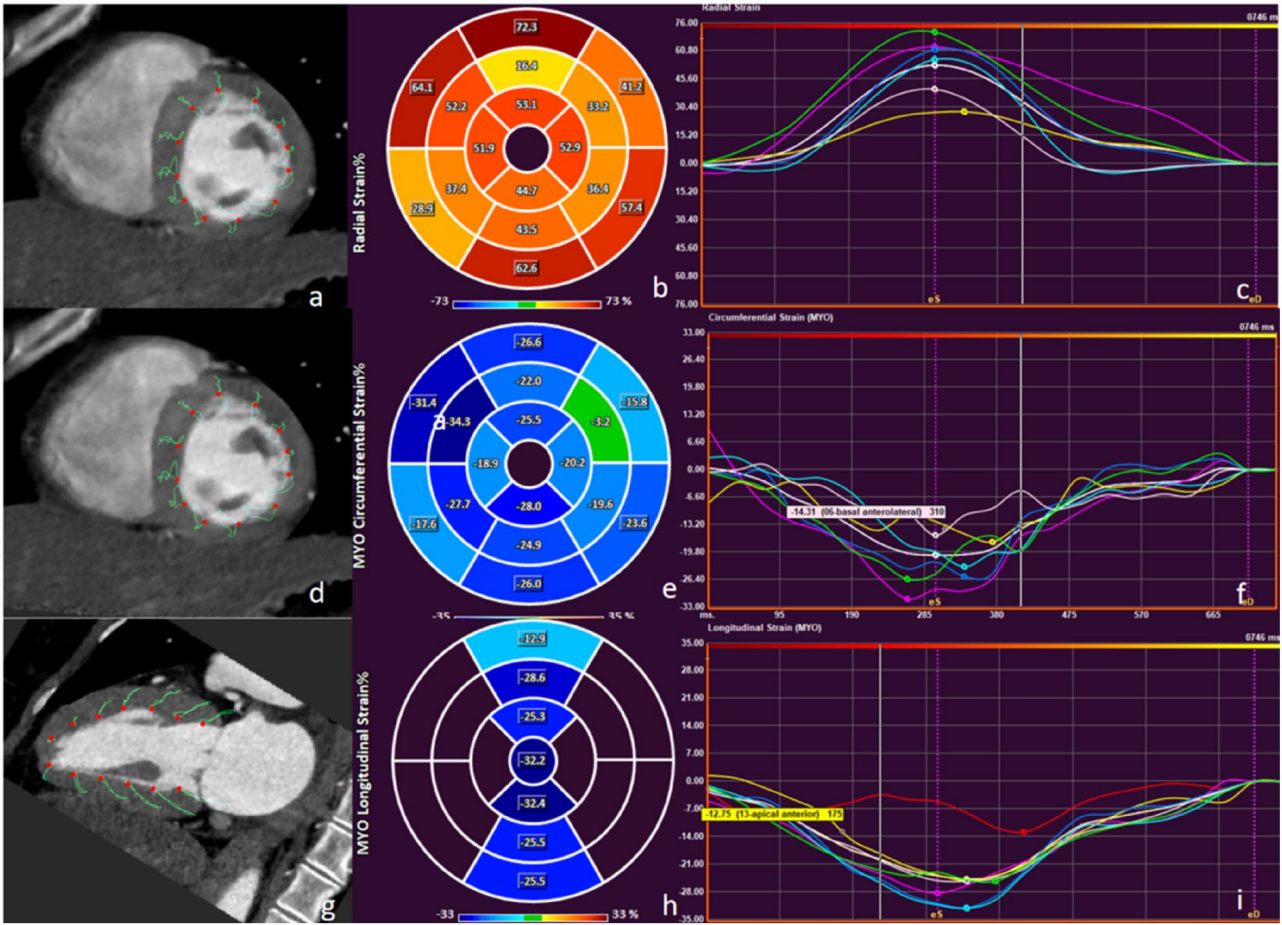
Schematic diagram of myocardial strain on post-processing software. (**a**, **d**, **g**) Images of different planes of the cardiac; (**b**, **e**, **h**) images of radial strain, circumferential strain and longitudinal strain; (**c**, **f**, **i**) curves of myocardial strain and time in the cardiac cycle.

The best diastolic and systolic reconstruction images were imported into the postprocessing workstation (syngo.via, Somatom Force, Siemens Healthineers), and coronary artery stenosis and CACs were analyzed by two experts with more than 10 years of experience in cardiovascular disease diagnosis. Obstructive CAD was defined as coronary CTA showing maximum diameter stenosis of ≥ 50% including groups III and IV. The disease groups were divided into 0 Agatston units (AU), 1–99 AU, 100–299 AU, and ≥ 300 AU according to the CACs. The commercial software (Medis suite version 3.0, Leiden, The Netherlands) automatically calculated the following cardiac functional parameters using data of 20 phases per cardiac cycle: LV end-diastolic volume (LVEDV), LV end-systolic volume (LVESV), stroke volume (SV), cardiac output (CO), LV ejection fraction (LVEF), global radial strain (GRS), circumferential strain (GCS), longitudinal strain (GLS). LV radial and circumferential strains were quantified on the short axis, whereas LV longitudinal strains were quantified on the long axis including 2-chambers, 3-chambers, 4-chambers and then averaged (Fig. 1).

### CMR data postprocessing and analysis

A commercial postprocessing software (Cvi42, Circle Cardiovascular Imaging, Calgary, AB, Canada) was used to analyze all CMR images. Myocardial strain analysis was performed by manually outlining the endocardial and epicardial borders of the LV at the end-diastolic and end-systolic phases.

### Repeatability

A total of 16 samples were randomly selected, and myocardial strain was independently measured by two radiologists with more than 5 years of experience in cardiovascular disease diagnosis. Additionally, one observer measured myocardial strain after 2 weeks.

### Statistical analysis

As reported in the previous literature^1^, the prevalence of CAD in adults was 5.6%. With a test level α of 0.05 and within a tolerance σ of 3%, the sample size was calculated on the formula: n = Z^2^_α_*P*(1−*P*)/σ^2^. The data were analyzed using Statistical Package for the Social Sciences Version 21.0. The normality of distributions for all continuous variables was assessed using the Shapiro–Wilk test. Continuous data with a normal distribution were expressed as x ± s, skewed data were expressed as median and upper and lower interquartile ranges, and categorical variables were expressed as frequencies (percentages). Differences between multiple groups were analyzed using one-way analysis of variance or the Kruskal–Wallis test. Additionally, Bonferroni and Tamhane’s methods were used for post hoc comparisons between two groups. Pearson’s and Spearman correlation coefficients were used to evaluate correlations between continuous variables as appropriate. Logistic regressions were used to analyze the effects of dichotomous variables. The Bland–Altman test was used to assess inter-method and intra- and inter-observer agreement. *P-*values of < 0.05 (two-tailed) were considered statistically significant.

### Ethical approval

The study was conducted according to the guidelines of the Declaration of Helsinki, and approved by the Institutional Review Board of Tongji Medical College, Huazhong University of Science and Technology (S 878; approval date on May 2019). All participants signed informed consent forms autonomously and voluntarily prior to participation.

## Results

### General clinical characteristics of the study population

A total of 237 patients with basic clinical information and coronary CTA examination data were enrolled. 546 patients were excluded from the study population (arrhythmias (n = 43), valvular heart disease (n = 45), cardiomyopathy (n = 54), congenital heart disease (n = 21), poor image quality (n = 35), a history of coronary artery reconstruction (n = 176), or insufficient clinical data (n = 172)). The general clinical characteristics and cardiac function parameters of each group are detailed in Table 1. The differences in age, sex, body mass index (BMI), CACs, LVEF, and LVESV were significantly different between groups.

**Table 1:** Clinical characteristics of the study population.

Variable	Control (n = 87)	Group I (n = 43)	Group II (n = 43)	Group III (n = 38)	Group IV (n = 26)	*P* Value
Age (years)	48.1 ± 11.1	55.7 ± 11.9*	57.8 ± 11.7*	63.7 ± 9.4*ab	61.5 ± 13.0*a	** < 0.001**
Males (n, %)	41 (47%)	23 (53%)	23 (53%)	24 (63%)	23 (88%)	**0.005**
BMI (kg/m2)	22.5 ± 2.7	24.1 ± 2.7*	25.0 ± 5.1	24.6 ± 5.7	23.5 ± 2.9	**0.006**
Smoker (n, %)	15 (17%)	8 (19%)	13 (30%)	11 (29%)	10 (38%)	0.140
Drinker (n, %)	19 (22%)	8 (19%)	7 (16%)	13 (34%)	8 (31%)	0.390
Hypertension (n, %)	0	12 (28%)	18 (42%)	19 (50%)	12 (46%)	** < 0.001**
Diabetes (n, %)	0	5 (12%)	11 (26%)	16 (42%)	9 (35%)	** < 0.001**
Dyslipidemia (n, %)	0	16 (37%)	13 (30%)	13 (34%)	9 (35%)	** < 0.001**
Family history of CAD (n, %)	15 (17%)	9 (21%)	10 (30%)	5 (13%)	6 (23%)	0.752
LVEDV (ml)	67.0 ± 10.9	66.2 ± 10.3	62.3 ± 9.2	62.6 ± 15.9	63.9 (53.3–106.5)	0.092
LVESV (ml)	29.0 ± 8.3	28.5 ± 7.7	28.3 ± 9.57	24.8 ± 7.3	31.4 (22.1–57.4) **c**	** < 0.001**
SV (ml)	39.2 ± 7.4	37.7 ± 6.7	36.5 ± 5.6	36.6 ± 9.7	32.3 (29.6–41.3)	0.485
LVEF (%)	57.9 ± 6.6	57.3 ± 8.1	57.2 ± 9.1	58.7 ± 6.9	48.5 (40.6–59.8) *c	** < 0.001**
CO (L/min)	2.7 ± 0.6	2.6 ± 0.5	2.6 ± 0.5	2.6 ± 0.8	2.5 (2.2–3.1)	0.902
Heart rate (HR) (beats/min)	65.5 ± 18.8	69.9 ± 9.8	69.6 ± 11.1	72.6 ± 13.6*	73.6 ± 14.0*	0.057
CACs (AU)	0	2.6 (0–12.4) *	33.5 (0–155.9) *	131.2 (14.6–316.2) *a	105.8 (6.0–467.4) *a	** < 0.001**

All data are expressed as the mean ± SD, and number of participants (without percentages), or median (interquartile range); BMI, body mass index; HR, heart beat; LVEDV, left ventricular end-diastolic volume; LVESV, left ventricular end-systolic volume; SV, stroke volume, CO, cardiac output; LVEF, left ventricular ejection fraction; *Compared with the control group, *P* < 0.05; a: compared with group I, *P* < 0.05; b**:** compared with group II, *P* < 0.05 ; c: compared with group III, *P* < 0.05.

Bolded: statistically significant P-values.

### LV myocardial strain in patients with different CAD-RADS levels

The myocardial strain in patients with different CAD-RADS levels is shown in Table 2. The LV GRS, GCS, and GLS differed significantly between CAD-RADS levels (*P* < 0.01). As the CAD-RADS level increased, GRS, the absolute values of GCS (|GCS|), and GLS (|GLS|) gradually decreased. In CAD-RADS level 1, a significant decreased in |GLS| was observed in the three LV global myocardial strains. The GRS, |GCS|, and |GLS| were the smallest in group IV and differed significantly between the control group and groups I–III (*P* < 0.01). GRS was significantly lower in group III than in the control group and group I.

**Table 2:** CT-derived global myocardial strain of different CAD-RADS levels.

Variable	Control group (n = 87)	Group I (n = 43)	Group II (n = 43)	Group III (n = 38)	Group IV (n = 26)	*P *Value
GRS	74.5 ± 15.2	74.1 ± 17.7	69.9 ± 15.6	66.8 ± 12.7*a	51.9 ± 20.3*abc	** < 0.001**
GCS	− 22.7 ± 3.01	− 21.6 ± 3.8	− 21.8 ± 3.8	− 21.5 ± 3.4	− 18.1 ± 4.5*abc	** < 0.001**
GLS	− 26.6 ± 3.2	− 23.9 ± 3.8*	− 23.3 ± 3.2*	− 22.0 ± 2.3*	− 19.1 ± 4.5*abc	** < 0.001**

All data are expressed as the mean ± SD, and all strains are expressed in percentages; GRS, global radial strain; GCS, global circumferential strain; GLS, global Longitudinal strain; *compared with control group, *P* < 0.05; a: compared with group I, *P* < 0.05; b: compared with group II, *P* < 0.05; c: compared with group III, *P* < 0.05.

Bolded: statistically significant P-values.

### Correlation between LV global myocardial strain and CACs

**Figure 2:**
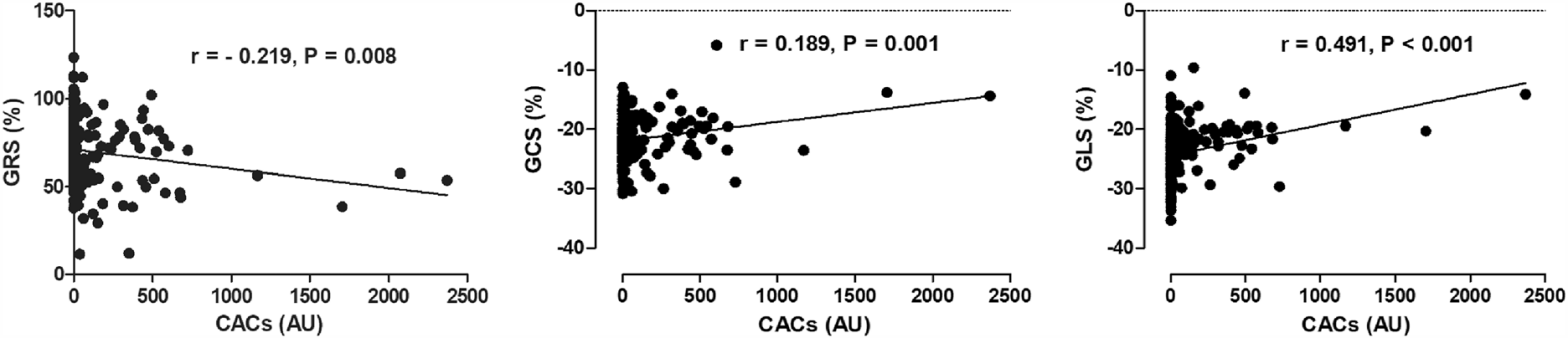
The correlation between left ventricular global myocardial strain and CACs. GRS: global radial strain; GCS: global circumferential strain; GLS: global longitudinal strain; CACs: coronary artery calcium scores.

GRS, GCS, and GLS were found to be significantly correlated with CACs (GRS: r =  − 0.219, GCS: r = 0.189, and GLS: r = 0.491, *P* < 0.01; Fig. 2). Myocardial strain for different levels of CACs is shown in Table 3. The absolute values of LV global myocardial strain decreased with increasing CACs levels. In the disease groups, GLS differed significantly between different CACs levels (*P* < 0.05).

**Table 3:** Global myocardial strain of different CACs levels.

Variable	Control group	Disease groups			
	CACs = 0 (n = 87)	CACs = 0 (n = 40)	1 ≤ CACs < 100 (n = 62)	100 ≤ CACs < 300 (n = 22)	CACs ≥ 300 (n = 26)
GRS	74.5 ± 15.2	70.2 ± 19.4	69.3 ± 15.7*	63.5 ± 18.6*	60.5 ± 18.9*ab
GCS	− 22.7 ± 3.01	− 21.1 ± 4.5	− 21.5 ± 3.4*	− 21.6 ± 3.9	− 19.5 ± 4.5*b
GLS	− 26.6 ± 3.2	− 23.5 ± 4.4*	− 22.8 ± 3.2*	− 21.2 ± 3.9*a	− 20.9 ± 3.2*ab

All data are expressed as the mean ± SD, and all strains are expressed in percentage GRS, global radial strain; GCS, global circumferential strain; GLS, global Longitudinal strain; *compared with control group, *P* < 0.05; a: compared with CACs = 0 on disease groups, *P* < 0.05; b: compared with 1 ≤ CACs < 100 on disease groups, *P* < 0.05.

Bolded: statistically significant P-values.

### LV global myocardial strain in obstructive and non-obstructive CAD

**Figure 3:**
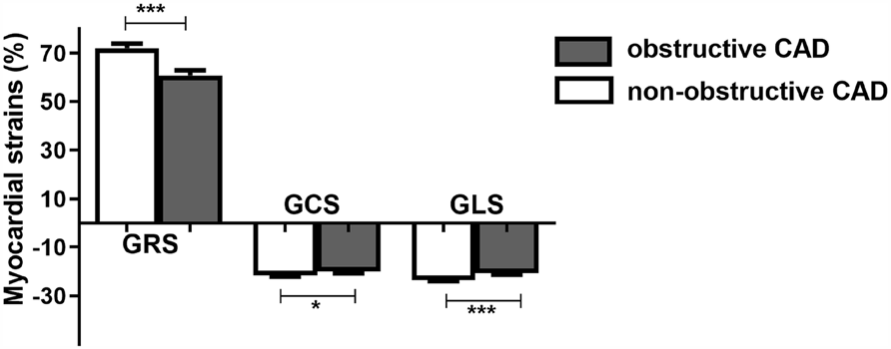
The differences of LV global myocardial strain between obstructive CAD and non-obstructive CAD; GRS: global radial strain; GCS: global circumferential strain; GLS: global Longitudinal strain; *P < 0.05, *** *P* < 0.001.

**Figure 4:**
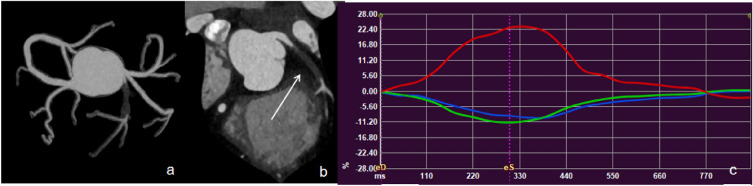
A 28-year-old man admitted with angina pectoris, CAD-RADS 5. (**a**) Image showed coronary artery calcification score of 0; (**b**) MPR revealed the anterior descending branch lumen obstruction (arrow); (**c**) curves of myocardial strain and time in the cardiac cycle. The peak systolic strains of the left ventricular myocardium were markedly decreased. Red, blue, green represents myocardial global radial strain, global longitudinal strain, and global circumferential strain respectively.

The differences in LV global myocardial strain between the two groups are shown in Fig. 3. The GRS, |GCS|, and |GLS| in the obstructive CAD group were significantly smaller than those in the non-obstructive CAD group. An example for LV myocardial strains based on CT in patient with CAD was shown in Fig. 4.

### Regression analysis

The results of the univariate and multivariate logistic regression analysis of the obstructive CAD group and the baseline clinical characteristics are summarized in Table 4. The independent determinants of obstructive CAD were age (*β* = 0.065, odds ratio [*OR*] = 1.067, *P* = 0.005), LVEF (*β* = 0.145, *OR* = 1.156, *P* = 0.047), and GLS (*β* = 0.232, *OR* = 1.261, *P* = 0.01).

**Table 4:** Univariate and multivariate regression analysis for obstructive CAD and the clinical and cardiac function parameters.

Variables	*β* Values	*Wals*	*P* Values	*OR* Values (95%CI)
Univariate analysis				
Age (years)	0.076	27.296	** < 0.001**	1.079 (1.048–1.11)
Female	− 1.005	9.792	**0.002**	0.366 (0.195–0.687)
BMI (kg/m^2^)	0.034	0.922	0.337	1.035 (0.965–1.11)
HR (beat/min)	0.027	5.598	**0.018**	1.028 (1.005–1.052)
Smoking	0.548	2.786	0.095	1.73 (0.909–3.292)
Dinking	0.62	3.512	0.061	1.858 (0.972–3.553)
Hypertension	1.499	21.843	** < 0.001**	4.478 (2.388–8.396)
Diabetes	1.773	23.189	** < 0.001**	5.887 (2.861–12.114)
Dyslipidemia	0.956	8.255	**0.004**	2.601(1.355–4.993)
History of CHD	− 0.164	0.184	0.668	0.849 (0.401–1.796)
LVEF (%)	− 0.038	5.674	**0.017**	0.963 (0.933–0.993)
GRS (%)	− 0.047	21.535	** < 0.001**	0.954 (0.935–0.973)
GCS (%)	0.152	12.193	** < 0.001**	1.165 (1.069–1.269)
GLS (%)	0.346	36.597	** < 0.001**	1.414 (1.264–1.582)
CACs (AU)	0.006	27.285	** < 0.001**	1.006 (1.004–1.009)
Multivariate analysis				
Age (years)	0.065	7.792	**0.005**	1.067 (1.019–1.116)
LVEF (%)	0.145	3.95	**0.047**	1.156 (1.002–1.335)
GLS (%)	0.232	6.582	**0.010**	1.261 (1.056–1.506)

BMI, body mass index; HR, heart beat; LVEF, left ventricular ejection fraction; GRS, global radial strain; GCS, global circumferential strain; GLS, global Longitudinal strain; CACs, coronary artery calcm score.

Bolded: statistically significant P-values.

### Repeatability analysis

**Figure 5:**
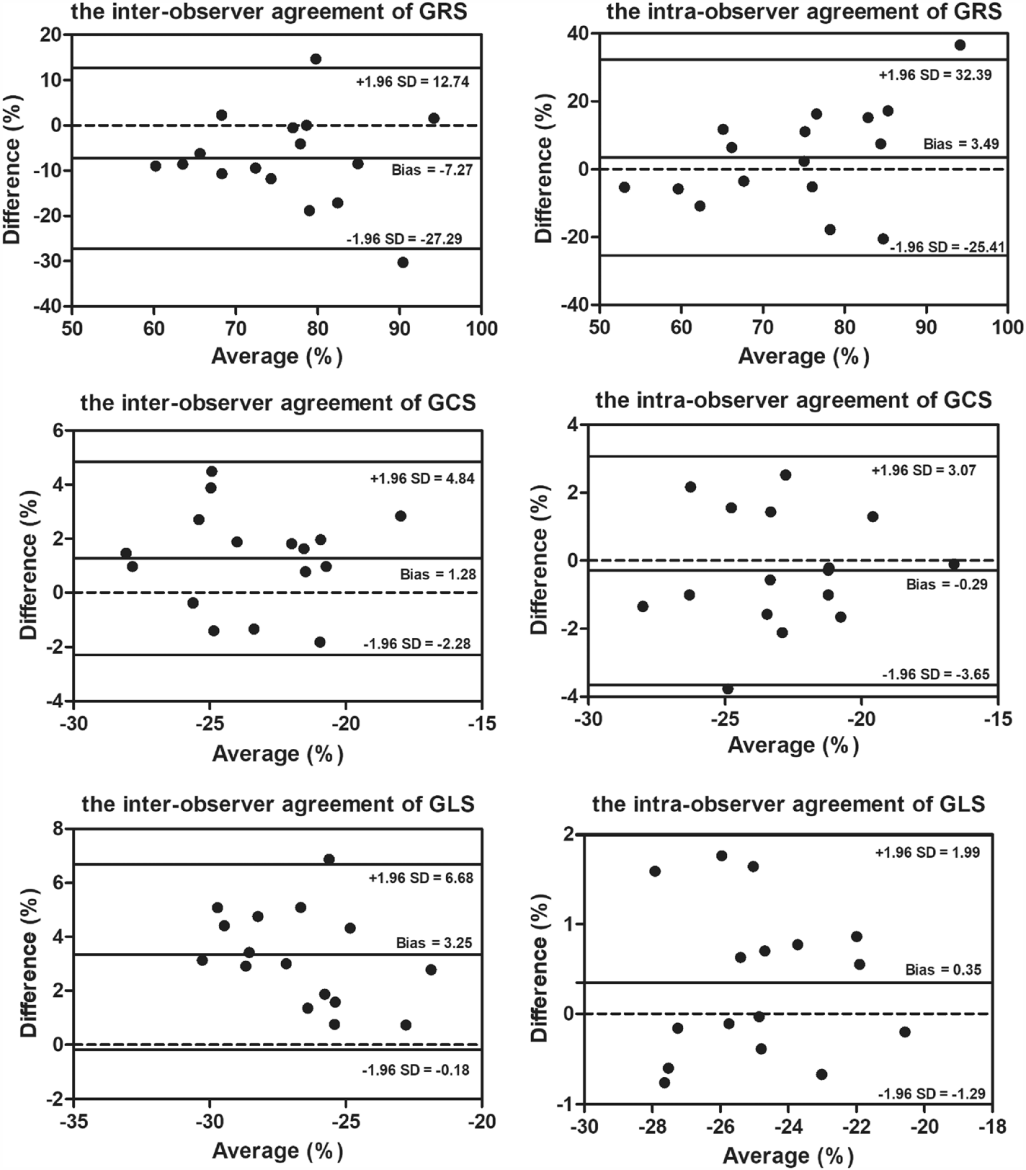
Bland–Altman analysis of the GRS, GCS, and GLS between intra-observer and inter-observer.

The Bland–Altman test was used for repeatability analysis between intra-observer and inter-observer agreement (Fig. 5). Only 6.25% (1/16), 12.5% (2/16), and 6.25% (1/16) of the points were outside the 95% confidence intervals on GRS intra-observer, GRS inter-observer, and GLS inter-observer agreements, respectively. All points were within 95% confidence interval on GCS intra-observer, GCS inter-observer, and GLS intra-observer agreements.

**Figure 6:**
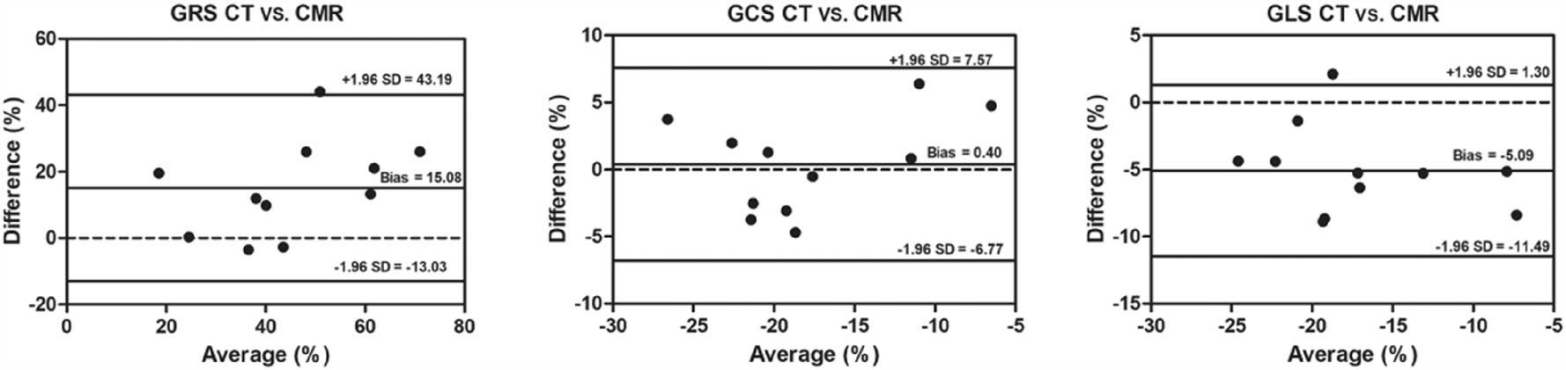
Bland–Altman analysis of the GRS, GCS, and GLS between CT and CMR.

Figure 6 shows the agreement between CT and CMR evaluation of strain, and all points were within the 95% confidence interval except for 1 point on GLS. The mean bias between the two methods was 15.08% for GRS, 0.4% for GCS and − 5.09% for GLS.

## Discussion

The CAD-RADS level depends on the severity of coronary artery stenosis, which influences the decision-making for the appropriate treatment option for patients. We evaluated the differences in CT-derived myocardial strain in patients with different CAD-RADS levels and investigated the relationship between cardiac CT-based myocardial strain and the degree of CACs and coronary stenosis. The results revealed that LV global myocardial strain based on CT gradually decreased as the CAD-RADS level increased. A significant correlation was found between LV myocardial strain and CACs.

We found significant differences in age, BMI, and CACs between groups. Age and BMI were greater in groups I–IV than that in the control group. Age is a non-adjustable risk factor for CAD, whereas obesity is a risk factor that can be adjusted by changing lifestyle habits^15^. The older the age and the higher the BMI, the greater the risk of CAD.

Compared with the control group, the heart rate (HR) was higher in groups III and IV, whereas LV global myocardial strain values were lower. Generally, HR is a predictor of cardiovascular and all-cause mortality in patients with cardiovascular diseases^16^. An increased resting HR leads to an increase in the risk of cardiovascular diseases, and its effect on cardiovascular mortality is independent of other cardiovascular risk factors^17^. Several studies have shown that increased resting HR is positively correlated with cardiovascular mortality^17–19^. HR is the main determinant of myocardial oxygen consumption and coronary blood flow^20^. In CAD, HR is a relevant factor affecting myocardial oxygen balance; an increase in HR causes a decrease in oxygen supply and an increase in oxygen demand, thereby leading to myocardial ischemia, decreased myocardial elasticity, and reduced myocardial strain values. An experimental study^21^ has revealed that there is a close linear relationship between myocardial blood flow and systolic function and that a decrease in local blood flow causes a corresponding decrease in systolic function.

Significant differences were found in LVEF between group IV and the control group and in GLS between groups I–IV and the control group. A significantly decreased GLS was observed before a significantly decreased LVEF as the CAD-RADS level increased. LVEF represents the percent change in LV chamber size and determines the entire cardiac systolic function, with limited regional myocardial systolic function. Compared with LVEF, GLS is more sensitive in CAD, which can be used as an early sensitive marker of clinically asymptomatic and mild LV insufficiency^22^. When ventricular wall motion abnormalities cannot be visually detected in the early stages, a local myocardial strain may help detect subtle ischemia-induced changes in myocardial function. Longitudinal myocardial fibers are mainly located in the subendocardium, and longitudinal strain mainly reflects the contractile function of subendocardial fibers, which are most vulnerable to ischemia^23^.

A previous study reported that LV myocardial strain measured using three-dimensional speckle-tracking echocardiography (3D-STE) decreases to varying degrees with increasing coronary artery stenosis^24^. Our study results were consistent with the findings of that study and showed that the absolute values of GRS, GLS, and GCS gradually decreased with an increase in the CAD-RADS level; this is because the coronary blood supply gradually decreases as the degree of coronary artery stenosis increases. This gradually leads to an imbalance in myocardial blood and oxygen supply and demand and the myocardium becoming ischemic, resulting in reduced myocardial elasticity and compliance and reduced myocardial strain values. Conversely, we evaluated LV global myocardial strain using the CAD-RADS classification, whereas the former study grouped patients according to intervals of 25% diameter stenosis. The variation of GRS in groups I and II and GCS within groups I–III were not statistically significant, suggesting that impaired LV short-axis motor function occurred later than long-axis function. In addition to the effect of myocardial fiber distribution, the radius of curvature of the circularly aligned middle layer fibers is smaller than that of the longitudinal fibers; thus, there is less myocardial stress and the appearance of functional abnormality is delayed^24^.

CACs is an independent predictor of future cardiovascular events^25^, which demonstrates its predictive value for symptomatic/asymptomatic CAD^26,27^. Kerut et al. used coronary artery calcification for future CAD risk assessment and found that the likelihood of CAD events in patients was related to the total plaque load in the coronary arteries^28^. They also found a significant correlation between myocardial strain and CACs. The higher the CACs, the lower the absolute value of global myocardial strains. We found that CACS and strain parameters were mildly to moderately correlated. This may be due to the fact that the majority of patients prospectively collected had CACs ≤ 300. In the future, an equal sample of patients will be collected within different graded ranges of calcification scores to reduce bias. Greenland et al. discovered that the incidence of future CAD events will increase when the CACs is greater than 300 AU^25^. In the present study, GRS, |GCS|, and |GLS| in the group with a CACs of ≥ 300 AU were significantly decreased, which is consistent with the findings of Erasmo et al.^29^. The risk of CAD prevalence is closely related to the calcification score, and CACs of 100 AU and 400 AU are considered the thresholds that distinguish hemodynamically significant low and high-risk groups for CAD prevalence, respectively^30^. Our results showed that myocardial strain values tended to decrease with an increase in disease risk, which was significantly lower in the control group than in the disease groups. Compared with CACs of 0 in the control group, |GLS| in CACs of 0 in the disease groups was significantly decreased, suggesting that GLS may help detect subclinical LV dysfunction earlier than CACs. A retrospective study with a large sample showed that a calcification score of 0 was associated with a very low risk of experiencing a coronary event in the future, regardless of the number of coronary risk factors^27^. This may be because the presence of noncalcified plaque causes severe lumen stenosis, resulting in a high CAD-RAD grade in the disease group with a CAC of 0.

The causes of INOCA include coronary microcirculation disorders, coronary artery spasms, and coronary artery coarctation. It lacks optimal treatment options in clinical practice. The present study showed a significant decrease in myocardial strain values of obstructive CAD, which is consistent with the results of a previous study^31^. Our study revealed that age, LVEF, and GLS are independent risk factors for obstructive CAD. The risk of obstructive CAD increased by 6.7% for each year of age and by 15.6% and 26.1% for each unit increase in LVEF and GLS, respectively. The findings revealed that a decreased GLS can be an indicator of non-obstructive CAD^32^. Moreover, GLS assessed at rest was an independent predictor of significant CAD in patients with suspected stable angina (*OR* = 1.25)^33^. That means in patients with low- and intermediate-risk chest pain, the addition of resting GLS to exercise echocardiography and conventional echocardiographic indicators improves the prediction of severe CAD. Severe coronary artery obstruction can result in myocardial infarction and ultimately heart failure. A prospective study found that GLS was independently associated with the risk of heart failure occurrence in patients with CAD^34^. It provided increased prognostic value to standard markers in patients with CAD with preserved or decreased LVEF.

This study has some limitations. First, obstructive CAD was not verified by coronary angiography. Invasive exams are more appropriate for patients with severe stenosis or a history of myocardial infarction. None of our patients had a history of myocardial infarction. CTA was used to exclude coronary artery disease, and most patients showed mild to moderate stenosis, the guidelines recommend that functional evaluation should be used as a priority in this group of patients^14^. Second, in the study, the mean heart rate was not statistically different between the groups, but few patients took beta-blockers when their heart rate was above 90 before performing the test, which may have affected the strain assessment. Third, to verify the consistency of CT and CMR, we randomly selected 11 patients for CMR examination. CMR was not performed on all patients because simultaneous examinations would likely add additional financial burden to patients and cause waste of medical resources. Finally, the study did not assess the regional LV myocardial strain in specific single coronary artery stenosis. However, studies related to single coronary lesions have been published by our team^7^. The focus of future research should be directed towards the application of CT-derived myocardial strain in the assessment of cardiomyopathy, as well as its potential to serve as a predictive tool for post-coronary revascularization outcomes.

In conclusion, we found a mild to moderate correlation between myocardial strains and CACs. The LV global myocardial strain absolute values based on CT gradually decreased as the CAD-RADS level increased. Age, LVEF, and GLS were identified as independent risk factors for obstructive CAD.

## Data Availability

The data presented in this study are available on reasonable request from the corresponding author.

## Abbreviations

BMI: Body mass index
CACs: Coronary artery calcium score
CAD: Coronary artery disease
CAD-RADS: Coronary artery disease-reporting and data system
CO: Cardiac output
CT: Computerized tomography
CTA: Coronary CT angiography
CMR: Cardiac magnetic resonance
ECG: Electrocardiograph
LV: Left ventricular
LVESV: Left ventricular end-systolic volume
LVEF: LV ejection fraction
GCS: Global circumferential strain
GLS: Global longitudinal strain
GRS: Global radial strain
SV: Stroke volume
